# Precopulatory acoustic interactions of the New World malaria vector *Anopheles albimanus* (Diptera: Culicidae)

**DOI:** 10.1186/s13071-019-3648-8

**Published:** 2019-08-01

**Authors:** Hoover Pantoja-Sánchez, Sebastián Gomez, Viviana Velez, Frank W. Avila, Catalina Alfonso-Parra

**Affiliations:** 1 Departamento de Ingeniería Electrónica, SISTEMIC, Universidad de Antioquia, Medellín, Antioquia 050010 Colombia; 2 Programa de Estudio y Control de Enfermedades Tropicales, PECET, Universidad de Antioquia, Medellín, Antioquia 050010 Colombia; 3 Instituto Colombiano de Medicina Tropical, Universidad CES, Sabaneta, Antioquia 055450 Colombia; 4 Max Planck Tandem Group in Mosquito Reproductive Biology, Universidad de Antioquia, Medellín, Antioquia 050010 Colombia

**Keywords:** Mosquito, Malaria, Harmonic convergence, Swarming, Mating

## Abstract

**Background:**

*Anopheles albimanus* is a malaria vector in Central America, northern South America and the Caribbean. Although a public health threat, *An. albimanus* precopulatory mating behaviors are unknown. Acoustics play important roles in mosquito communication, where flight tones allow males to detect and attract potential mates. The importance of sound in precopulatory interactions has been demonstrated in *Toxorhynchites brevipalpis*, *Aedes aegypti*, *Culex quinquefasciatus* and *Anopheles gambiae*; convergence in a shared harmonic of the wing beat frequency (WBF) during courtship is thought to increase the chance of copulation. To our knowledge, *An. albimanus* precopulatory acoustic behaviors have not been described to date. Here, we characterized *An. albimanus* (i) male and female flight tones; (ii) male–female precopulatory acoustic interactions under tethered and free flight conditions; and (iii) male-male acoustic interactions during free flight.

**Results:**

We found significant increases in the WBFs of both sexes in free flight compared to when tethered. We observed harmonic convergence between 79% of tethered couples. In free flight, we identified a female-specific behavior that predicts mate rejection during male mating attempts: females increase their WBFs significantly faster during mate rejection compared to a successful copulation. This behavior consistently occurred during mate rejection regardless of prior mating attempts (from the same or differing male). During group flight, males of *An. albimanus* displayed two distinct flying behaviors: random flight and a swarm-like, patterned flight, each associated with distinct acoustic characteristics. In the transition from random to patterned flight, males converged their WBFs and significantly decreased flight area, male-male proximity and the periodicity of their trajectories.

**Conclusions:**

We show that tethering of *An. albimanus* results in major acoustic differences compared to free flight. We identify a female-specific behavior that predicts mate rejection during male mating attempts in this species and show that male groups in free flight display distinct flying patterns with unique audio and visual characteristics. This study shows that *An. albimanus* display acoustic features identified in other mosquito species, further suggesting that acoustic interactions provide worthwhile targets for mosquito intervention strategies. Our results provide compelling evidence for swarming in this species and suggests that acoustic signaling is important for this behavior.

**Electronic supplementary material:**

The online version of this article (10.1186/s13071-019-3648-8) contains supplementary material, which is available to authorized users.

## Background

Malaria is caused by *Plasmodium* parasites that are transmitted through the bites of infected *Anopheles* mosquito vectors and represents a major disease that afflicts the inhabitants of tropical countries. Although most lethal malaria cases are concentrated in sub-Saharan Africa [1], numerous cases of the disease occur in South America [2]. In Colombia, an estimated 12 million people live in malaria endemic areas and are at risk of infection [3]. In 2017, ~53,000 cases of malaria were reported in Colombia [4], with the majority originating in areas near the Atlantic and Pacific coasts. Despite the implementation of various control methods, which include indoor and outdoor insecticide spraying and the use of insecticide impregnated bednets, malaria continues to be a major public health issue in Colombia [5, 6].

*Anopheles albimanus* (subgenus *Nyssorhynchus*) is a vector of malaria throughout Central America, the northern portion of South America and the Caribbean [7]. In Colombia, *An. albimanus* is one of three primary vectors of malaria [7]. Despite its status as an important vector in the Americas, there exists a large gap in our knowledge of the behavior, ecology and biology of this species, making the development and/or improvement of control methods difficult. Much of our understanding of anopheline reproduction is inferred from studies in *An. gambiae* (subgenus *Cellia*), whose post-mating reproductive biology differs substantially from *An. albimanus* [8].

Mosquito vectorial capacity is dependent on biological factors that include population density, female blood-feeding rate and female longevity [9], each related to successful reproduction [8, 10, 11]. Thus, processes of reproduction offer promising targets to control mosquito vectors and, by extension, the diseases they spread [12–14]. Contemporary mosquito control methods include the mass release of transgenic or sterile males to suppress native mosquito populations [15, 16]. Therefore, mass-released males need to compete with wild males for mating opportunities, which require males to detect and successfully interact with females. In *Aedes* [17, 18], *Culex* [19] and *Anopheles* species [20, 21], acoustic signals are used by males to locate and recognize females. Males detect nearby flying females of the same species by the sound of their wing beat frequency (WBF) [20, 22–24], pursue them, and appear to acoustically interact with them at a local scale [25, 26] prior to copulation. It has also been suggested that females use acoustic signals as an indicator of male fitness [27, 28].

In several *Anopheles* species, the initiation of reproductive behavior begins when males form a swarm [29]. In African vectors such as *An. funestus*, *An. gambiae* and *An. arabiensis*, swarm formation begins when a few males begin to fly simultaneously [30–33]; additional males rapidly join the flight, increasing the swarm size and density [29, 34]. At the initiation of swarm formation, males fly in a non-specific circular motion before forming a more tightly patterned, cohesive group [35]. Once formed, males either mate with females that penetrate the swarm or depart the swarm to mate with a female flying nearby [35]. To date, acoustic interactions between swarming males have not been described for *Anopheles* species. However, cohesive acoustic behavior between multiple tethered *Ae. aegypti* males has been observed [36]: male groups exhibit similar flight tones, a phenomenon more evident in larger groups [36], although both frequency convergence and divergence behaviors are observed between pairs of tethered *Ae. aegypti* males in close proximity [37], showing how variable such interactions can be. Frequency divergence has also been reported between tethered *Cx. quinquefasciatus* males [19]. Characterization of male-male acoustic interactions may be key in understanding swarm formation and swarm cohesion.

Studies describing acoustic interactions between opposite-sex mosquitoes are more numerous. In several species, female flight tones act as a mating call that attracts males of the same species [23, 38, 39]. In experiments with tethered *Ae. aegypti* [17], *Cx. quinquefasciatus* [19] and *An. gambiae* [20, 21], males and females modulate their WBFs to match each other during courtship, a phenomenon known as harmonic convergence. Such convergence does not happen in the WBF of the flight tones but instead in a shared harmonic [17, 21]. An additional acoustic behavior, characterized by the rapid frequency modulation (RFM) of the WBF, was first detected in *Cx. quinquefasciatus* [25] and later described in *An. coluzzi* [40], *An. gambiae* [40] and *Ae. aegypti* [26]. RFM is observed after a steep increase of the male WBF when in close proximity to a tethered female or an artificial tone source [23, 38, 39]. In all species studied, RFM occurs prior to copulation. In *Ae. aegypti*, RFM coincides with initial contact of a female by a courting male and ends prior to copulation or separation of the pair [26]. Moreover, RFM and harmonic convergence appear to be independent events [25, 26, 40].

To date, flight acoustics and male–female pre-mating acoustic interactions have not been investigated in *An. albimanus*. Furthermore, it is unknown if males of this species swarm in the wild. To gain insight into *An. albimanus* precopulatory acoustic behaviors, we first characterized flight acoustics in this species by determining male and female WBFs when tethered and in free flight. We observed a significant increase in the WBF of each sex when in free flight compared to when tethered. We next characterized male–female acoustic interactions when tethered and in free flight during courtship. We found that 79% of tethered couples display harmonic convergence. Using criteria established for tethered couples, we did not detect harmonic convergence in free fight, possibly due to the brevity of mating attempts (1.38 ± 0.32 s) and the absence of paired flight after couples made contact (when harmonic convergence likely occurs; [26]). However, we observed that during mate rejection, females increase their WBFs significantly faster than those that mate. Finally, using audio and visual analysis, we show that male groups in free flight change flight trajectories and match flight tones during a stereotypic, patterned flight, which could be suggestive of swarm-like behavior in *An. albimanus*. Our results represent important first steps toward dissecting the subtle, pre-mating behavioral interactions that occur in this important malaria vector.

## Methods

### Mosquito rearing

*Anopheles albimanus* were obtained from the colony maintained at Programa de Estudios y Control de Enfermedades Tropicales (PECET), Universidad de Antioquia, Colombia. These mosquitoes were originally collected in Santa Rosa, Bolivar, Colombia, and have been kept in colony since 1995. Mosquitoes were reared and maintained in a walk-in climate chamber at 27 °C and 80% relative humidity (RH) under a 12:12 h light:dark photoperiod. Eggs were collected in 473-ml cups filled with dH_2_O. Upon hatching, larvae were individually transferred to trays (~60 per tray) containing 0.5 l of dH_2_O. Larvae were fed daily with 20 mg of fish food (Tetracolor; Tetra, Melle, Germany). Pupae were individually transferred to 15-ml vials to ensure virginity. Upon eclosion, adults were separated by sex, transferred to 4-l plastic cages, and held in same-sex groups until experiments commenced. Mosquitoes had access to 20% sucrose *ad libitum*. We used 4–6-day-old adults in all experiments.

### Tethered mosquitoes

#### Audio recording setup

Recordings were performed in a soundproof chamber that contained two particle velocity microphones (NR-23158-000, Knowles; Itasca, IL, USA). Microphone signals were amplified and digitalized by an USB audio interface (M-Track Quad Four Channel Audio; M-Audio, Cumberland, USA). Mosquito flight tones were processed using Matlab (R2016a, The Mathworks Inc, Natick, USA) at a sample rate of 11025 Hz/24 bits.

#### Experimental procedures

Males and females were recorded to determine their individual WBF distribution. Virgin mosquitoes were anesthetized on ice and tethered to a human hair using a cyanoacrylate-based adhesive (Super Pega Infinita, Medellin, Colombia). The hair was attached on one end to an insect pin while the opposite end was attached to the pronotum (dorsal section of the thorax) as in [27]. Before recording, the position of the hair was checked to ensure natural wing movement. To record flight tones, mosquitoes were placed ~1 cm above a single particle velocity microphone. When necessary, flight was triggered by blowing or by gently moving the legs of the individual.

Using tethered individuals as described above, male–female interactions (couples) were recorded to determine if *An. albimanus* display the precopulatory behavior of harmonic convergence [17]. One mosquito was placed 1 cm above one microphone and kept stationary. A tethered individual of the opposite sex, placed ~1 cm above a second microphone, was brought in and out of the near field of the first, stationary mosquito for intervals of 10 s with 5 s of rest between intervals. After repeating 3 times, the positions of the individuals were switched. Wing length of males and females were measured as previously described [41] to estimate individual size. All experiments were performed at 26 ± 2 °C. Tone analysis was performed using recordings that had > 10 s of continuous flight.

#### Signal analysis

Since mosquito flight tone has been described as a harmonic sound [4], a sinusoidal-harmonic model was used to extract the WBF information of the recorded signals. From the spectrogram [fast Fourier transform (FFT) length of 4096 points, hamming window of 80 ms and 50% overlapping], we extracted the WBF by tracking the lower frequency peak of the spectrogram that persisted for at least 1 s and fulfilled two conditions: between consecutive windows, the frequency and power did not change more than ± 20 Hz and ± 10 dB FS, respectively. For individual flights, we analyzed WBF mean and WBF range of both sexes. For male–female interactions, we identified harmonic convergence as in Aldersley et al. [37]. Comparing the extent of frequency modulation and frequency distribution prior to and during interactions, we identified events where there was an active modulation of the WBF in response to the conspecific flight tone. To label convergence events as positive or negative, we used a time-varying fundamental frequency ratio between females and males (♀WBF/♂WBF). For instance, a ratio of 0.667 means that the female’s third harmonic is converging with the males second. We set a positive convergence as an event of duration greater than 1 s, in which the convergence ratio was within an interval of tolerance ± 1.5%. The time between individual introduction and the onset of convergence was noted as latency [21]. Analysis was performed using Matlab (R2016a, The Mathworks Inc, Natick, USA)

### Free-flying mosquitoes

#### Audio and visual recording setup

Audio and video of free-flying mosquitoes were simultaneously recorded in an experimental arena (20 × 20 × 25 cm transparent plastic box) placed within a sound-proof chamber. The experimental arena contained four electret microphones (Knowles FG-23329-C05), three placed on the side and back walls and one on the bottom of the box. Microphone signals were amplified by an operational amplifier (INA128; Texas Instruments, Dallas, USA) and digitalized by a 16-channel digital-analog converter (779676-01; National Instruments, Austin, USA) at a sample rate of 11025/16 bits. For video recording, a webcam (HD Pro Webcam C920; Logitech, Lausanne, Switzerland) was placed 30 cm in front of the arena to follow mosquito movement.

#### Experimental procedures

Three types of recordings were performed. (i) We recorded individual males and females inside the arena (*n* = 30 for each sex). (ii) Male–female acoustic interactions were recorded by introducing two males into the arena followed by one female. Flight tones were continuously recorded until copulation was observed or the individuals stopped flying. Mosquitoes were removed after a successful copulation. Successful copulations were verified by dissecting females to determine insemination status (i.e. presence/absence of sperm in the spermathecae). A mating attempt was counted as successful if a couple locked genitalia for more than 5 s and sperm was transferred to the female. An attempt was considered a rejection when the male and female genitalia did not come into contact or the female did not allow the male to lock genitalia. As females often rejected courting males, we recorded 167 total mating attempts from 42 different females. Recordings from 9 females were discarded, as it was not possible to clearly identify the frequency components of each sex. (iii) Acoustic interactions of male groups were recorded by introducing 8 mosquitoes into the arena 5 h before recording started. Although 8 mosquitoes were used in every experiment, only groups of 2, 3 and 4 mosquitoes flying simultaneously were analyzed. For each experiment, recordings (2.5 h in length) were segmented into 10 min sections. When a specific flying behavior was observed visually, unique fragments of 5 s were used in our analysis (for the respective number of males flying). All recordings were performed at 26 ± 2 °C between 16:00 and 18:30, corresponding to the 2 h prior to sunset.

#### Audio signal analysis

Signals from each of the four microphones were used to extract frequency components of the flight tones. By obtaining the cross spectrum among the microphones (FFT length of 4096, window length 0.08 s, 50% overlapping) [42], we generated a high order spectrogram which was used to analyze the signals continuously, regardless of the distance between mosquitoes and microphones. Depending on the interaction analyzed, we extracted different frequency features from the spectrograms. (i) For male and female individual flight, the WBF distribution and range was determined. (ii) For male–female interactions, we visually selected relevant steps of mating and analyzed the WBF ratio (♀WBF/♂WBF) 1–1.5 s prior to a mating attempt, the rate of WBF increase during a mating interaction and the response latency of the WBF increase of the female following that of the male. The change in frequency (ΔF = F_max_ − F_0_) was measured as the difference between the initial frequency at the onset of the increase (F_0_) and the maximum frequency reached (F_max_). For females, however, the change in frequency was divided by two, as measurements where extracted from the second harmonic (ΔF = (F_max_ − F_0_)/2). The duration of the increase was measured by using the time at the same two points (Δt = t_max_ − t_0_). Finally, we calculated the rate of increase (ΔF/Δt) of each sex. (iii) For male groups, we measured the average difference between the highest and the lowest WBFs, referred hereafter as frequency difference.

#### Video analysis

Audio recording from the microphone integrated to the camera was used to synchronize audio and video. We used a 1 kHz pure tone to synchronize the camera and electret microphones. Once audio and video were synchronized, we extracted 5 s video fragments from recordings in which the specific behavior was observed. Each fragment was segmented into individual frames and each frame converted to grayscale and adjusted through the equalization of its histogram to improve the contrast of the image. Finally, for each frame, X and Y coordinates of each mosquito were obtained manually to reconstruct flight trajectories using Matlab (R2016a, The Mathworks Inc, Natick, USA). Trajectories were represented by ellipse due to the natural flight of the individuals, following previous methodologies [43]. In brief, ellipse area was calculated to represent the area covered by the movement of each mosquito. The center of the ellipse corresponded to the mean position of each mosquito trajectory (the X and Y coordinates) during the 5 s analyzed. The horizontal and vertical diameters of each ellipse were calculated as 4 times the standard deviation of each trajectory point of each individual. The average distance of the centroids of each ellipse in relation to the others was calculated to assess the extent of the aggregation of the group. Finally, the loop period of each trajectory was calculated to assess the periodicity of the movement, as in Gibson [43].

### Statistical analysis

For tethered mosquitoes, we performed a linear regression analysis to determine the relationship between wing length (as a size index) and WBF. To compare frequency distributions and ranges of tethered and free-flying individuals, we performed a t-test and a factor analysis. As factors, we evaluated sex and recording method. Residuals of t-tests and factorial models were tested for normality, homogeneity of variance and independence using Shapiro-Wilks, Bartlett and Durbin-Watson tests, respectively. Aspects influencing male–female interactions during free flight trials were assessed using the non-parametric Mann–Whitney U-test. The frequency difference broadcast by male groups and the differences between the characteristics of flight trajectories were assessed using a Mann–Whitney-U test. Results are presented as mean ± SEM. Sample sizes are indicated for each experiment.

## Results

### Flight tones of tethered and free-flying *An. albimanus* males and females

**Fig. 1:**
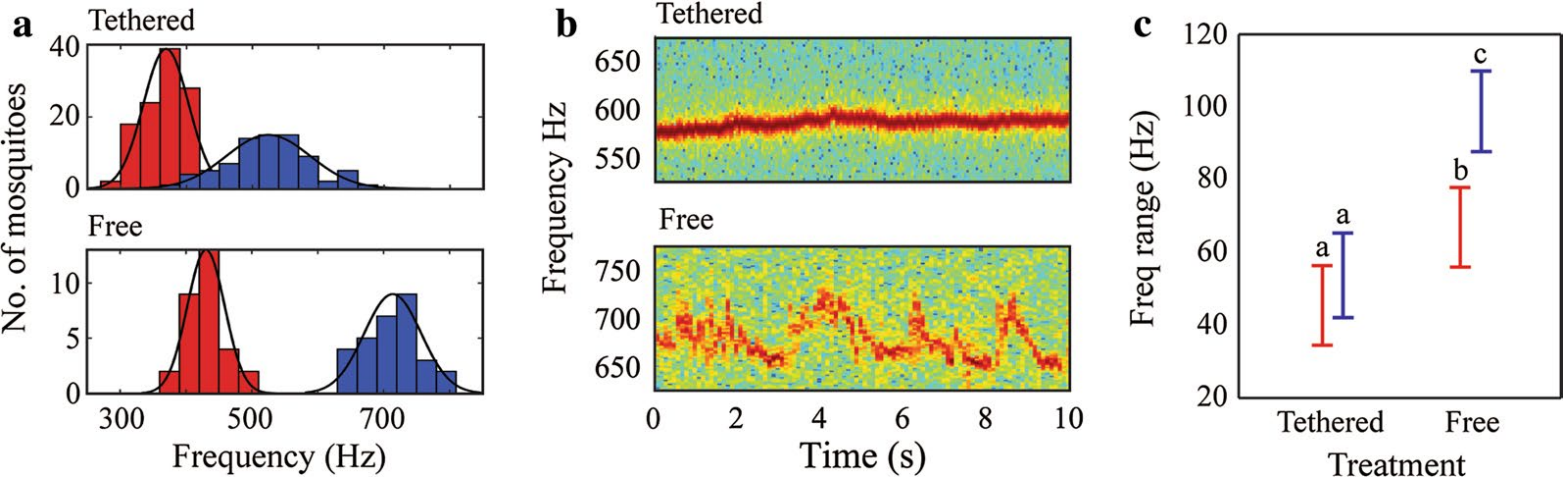
Flight tones of tethered and free-flying *An. albimanus* individuals. **a** Top: WBF distribution of tethered males (*n* = 78, blue) and females (*n* = 116, red). Bottom: WBF distribution of free-flying males (*n* = 30, blue) and females (*n* = 30, red). **b** Spectrograms of a representative tethered (top) and free-flying (bottom) male. **c** WBF range analysis of males (*n* = 30) and females (*n* = 30) when tethered or in free flight. There were significant effects for both sex and treatment. Vertical bars denote 0.95 confidence intervals and letters indicate significant differences for a *post-hoc* Tukey test with 0.95 confidence

To characterize *An. albimanus* flight tones, virgin males and females were individually recorded while tethered or in free flight. When tethered, we observed a WBF of 524.11 ± 63.73 Hz (mean ± SEM, *n* = 78) for males and 368.91 ± 34.28 Hz (*n* = 116) for females (Fig. 1a). There was a significant difference in the WBF between males and females (t-test: *t*_(192)_ = − 21.94, *P* < 0.01). By measuring the wing length of males (2.78 ± 0.02 mm) and females (2.89 ± 0.02 mm), we did not find evidence of a relationship between individual size and WBF (Linear regression; males: *R*^2^ < 0.01, Pearson’s *r*_(78)_ = 0.06, *P* = 0.58; females: *R*^2^ = 0.03, Pearson’s *r*_(116)_ =  − 0.20, *P* = 0.05; Additional file 1: Figure S1a). Thus, size did not appear to significantly influence the WBF in tethered individuals of either sex.

Because various studies of insect flight have reported that WBFs of tethered individuals differ compared to individuals in free flight [4, 25, 44, 45], we sought to determine how *An. albimanus* acoustics might differ in this regard by examining mosquitoes in flight. Males and females were individually introduced into a soundproof box and their flight tones recorded. During free flight, we observed a WBF of 713.76 ± 43.16 Hz (*n* = 30) for males, and 430.34 ± 28.48 Hz (*n* = 30) for females (Fig. 1a). A significant increase in flight tone was observed for individuals in free flight compared to tethered individuals (t-test; males: *t*_(106)_ = − 9.03, *P* < 0.01; females: *t*_(144)_ = − 15.00, *P* < 0.01). Similar to tethered individuals, we did not observe a strong relationship between mosquito size (males: 2.83 ± 2.02 mm; females: 3.05 ± 220.53 mm) and WBF (Linear regression; males: *R*^2^ = 0.12, Pearson’s *r*_(45)_ = 0.35, *P* = 0.02; females: *R*^2^ < 0.01, Pearson’s *r*_(54)_ = 0.03, *P* = 0.81; Additional file 1: Figure S1b).

When analyzing each spectrogram, we noted that the tones of individuals were characterized by larger modulations of the WBF when in free flight compared to when tethered (Fig. 1b). To evaluate the effect of tethering on frequency modulation, we randomly selected a sample of 30 tethered males and 30 tethered females to compare them with 30 males and 30 females in free flight by performing a factorial ANOVA, using the recording method and sex as factors. Sex (factorial ANOVA: *F*_(1, 116)_=13.1, *P* < 0.01) and tethering (factorial ANOVA: *F*_(1, 116)_ = 37.57, *P* < 0.01) each affected the range of modulation, and an interaction between these two factors was observed (factorial ANOVA: *F*_(1, 116)_ = 4.66, *P* = 0.03). Thus, when males and females are tethered, the range of their WBF modulation is similar (males: 53.33 ± 7.89 Hz; females: 45.25 ± 3.14 Hz) but is significantly increased (males: 99.10 ± 5.75 Hz; females: 67.17 ± 4.67 Hz) during free flight (Fig. 1c).

### Harmonic convergence between tethered *An. albimanus* males and females

**Fig. 2:**
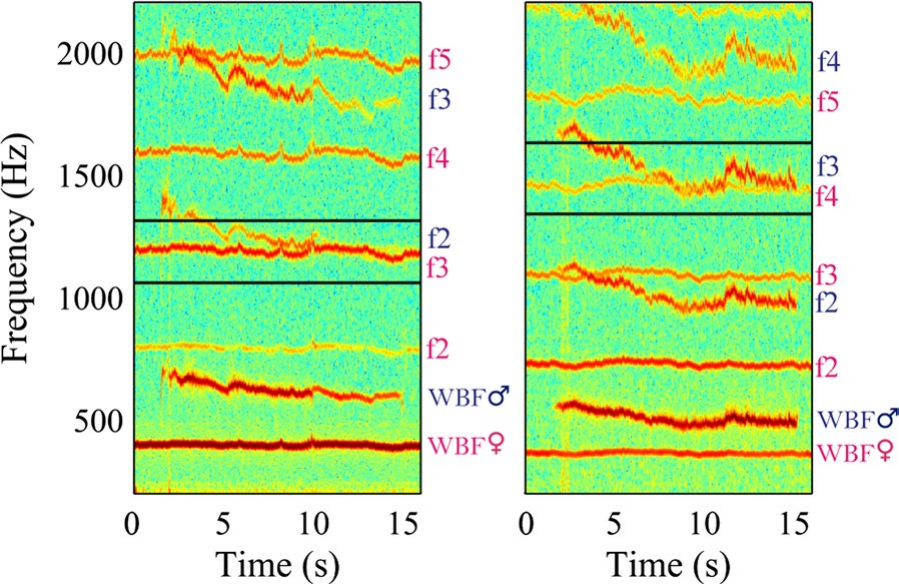
Harmonic convergence between tethered couples. Spectrograms of males and females are shown, displaying the WBF and harmonics. Pink labels indicate female harmonics, blue labels indicate male harmonics. Black rectangles highlight harmonic convergence between female f3 and male f2 (left) and female f4 and male f3 (right)

Since harmonic convergence has been previously described using tethered mosquitoes of other species [17, 19, 20], we characterized harmonic convergence using tethered male and female *An. albimanus*. We observed harmonic convergence in 41 of 52 couples (78.8%). By analyzing the ratio of the female WBF with respect to the males’, we observed two distinct groups where convergence was observed: in 21 couples (40.4%), the female’s third harmonic converged with the male’s second (Fig. 2, left panel), while in 20 cases (38.5%), the female’s fourth harmonic converged with the male’s third (Fig. 2, right panel). The average time of convergence was 2.00 ± 0.81 s and the response latency ranged from 4 to 13 s. The remaining couples did not match harmonics. We did not detect a relationship between the ratio of couple size (♂size/♀size) and the ratio of convergence (t-test: *t*_(40)_ = 0.68, *P* = 0.49): couples where the female’s third harmonic converged with the male’s second had a size ratio of 0.954 ± 0.015; couples where the female’s fourth harmonic converged with the male’s third had a size ratio of 0.942 ± 0.007.

### Male–female precopulatory acoustic behaviors during free flight

To characterize mosquito flight tones prior to copulation, we examined male–female acoustic behaviors during mating attempts. Male mating attempts were determined by visual and audio analysis: a male “chasing” a female [25, 40] was classified as the onset of a mating attempt. From 33 different females, 134 mating attempts were analyzed. Mating attempts resulted in either a copulation (i.e. the female was inseminated) or a mating rejection; 12 of 33 females mated. The duration of a mating interaction was brief (1.38 ± 0.32 s; *n* = 33). Ten of the mated females rejected a male at least once, while two mated after the first mating attempt. All mated females copulated within 1–5 min after introduction to the arena. Females that did not mate in our assays rejected males 1–9 times, with females that rejected once (*n* = 3) not flying the remainder of the assay. To compare mating attempts that ended in a successful copulation or a rejection, we examined audio recordings of the 12 interactions that resulted in a successful copulation and randomly selected one mating attempt from each of the 21 females that did not mate in order to identify male- or female-specific acoustic behaviors that occur immediately prior to *An. albimanus* copulation or mate rejection.

**Fig. 3:**
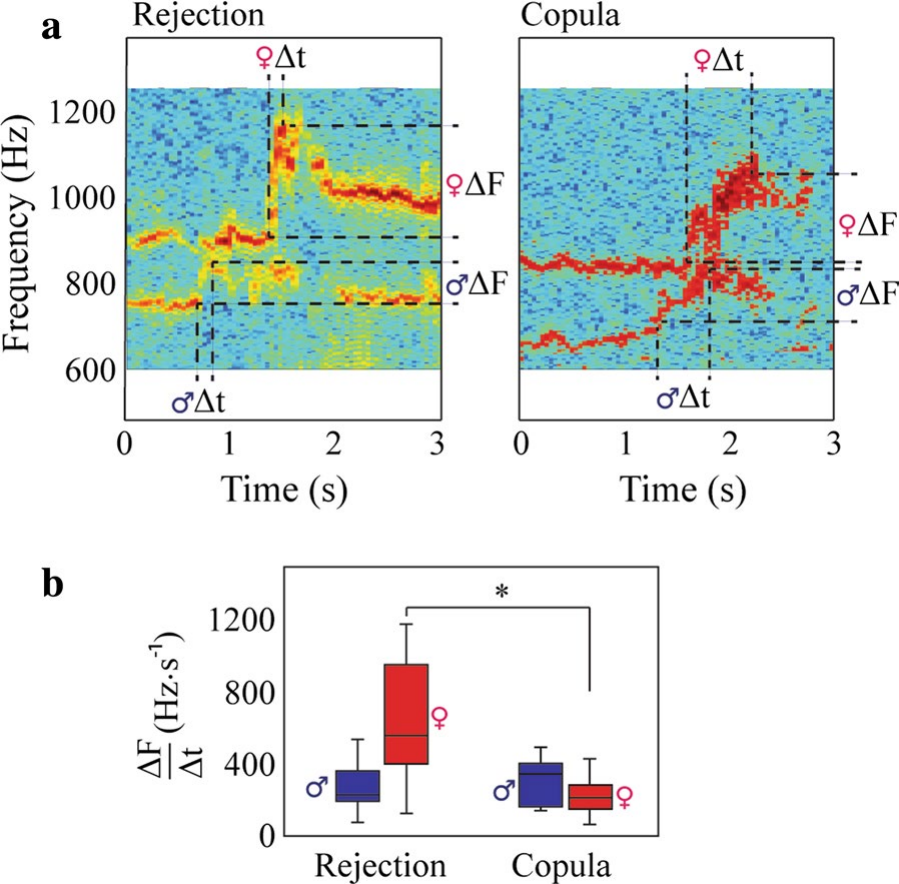
Acoustic analysis of free-flying mosquito during courtship. **a** Representative spectrograms of a mating attempt that resulted in a mating rejection (left) or a copulation (right). The spectrograms show the males fundamental frequency (WBF) and the females’ second harmonic (analysis was performed using the WBFs of each sex). The change in WBF (ΔF) and the time interval between the initial WBF increase and the maximum frequency reached (Δt) is shown for each sex. **b** Comparison of the rate of frequency increase (ΔF/Δt) for male–female interactions that ended in a rejection (*n* = 21) or copulation (*n* = 12). *Signifies a significant difference for a Mann–Whitney U-test (*P* < 0.01)

We consistently observed that all mating attempts, regardless of outcome, were characterized by an increase in male WBF followed by an increase in female WBF (Fig. 3a; Additional file 2: Video S1). The increase in the WBF of the male coincided with the onset of the female chase, while that of the female coincided with her flying away from the approaching male. After increasing their WBFs, males and females rapidly modulated their frequency until couples departed or stopped flying. Although these oscillations appear to be similar to those reported in other species [25, 26, 40], it was not always possible to observe this behavior in every mating attempt as 25% of the couples that copulated landed upon making contact. The time interval of rapid frequency oscillation after reaching maximum WBF ranged between 0.34–1.38 s and 0.18–1.56 s for males and females, respectively.

Using the criteria for tethered pairs in Aldersley et al. [37], we did not detect harmonic convergence in free-flying pairs. The brevity of the mating interaction, the WBF range of individuals in free flight, and that several couples ceased flying upon contact suggest that our methods were not sufficient to detect this phenomenon. However, we analyzed the latency of the response of the female, the female/male WBF ratio prior the interaction and the WBF increase during the mating attempt to determine if these characteristics influenced the outcome of a mating attempt. The latency of the females’ WBF increase in response to that of the male did not significantly differ between interactions that resulted in a copulation or a rejection (copulation: 0.375 ± 0.074 s; rejection: 0.542 ± 0.047 s; Mann Whitney U-test: *U*_(12, 21)_ = 78, *Z* = − 1.79, *P* = 0.07). The female/male frequency ratio average (♀WBF/♂WBF) 1–1.5 s prior to a copulation, before any observable male–female interaction occurred, was similar for mating attempts that resulted in copulation (♀WBF/♂WBF: 0.595 ± 0.024) or rejection (♀WBF/♂WBF: 0.596 ± 0.024; Mann–Whitney U-test: *U*_(12, 21)_ = 125, *Z* = − 0.03, *P* = 0.97) and did not influence mating outcome. Finally, to analyze the WBF increase during the mating attempt, we measured the change in WBF (ΔF) and the time interval between the initial WBF increase and the maximum frequency reached (Δt), allowing us to calculate the WBF rate of increase (ΔF/Δt) of both sexes during this interaction (Table 1, Fig. 3a). In males, we found that the rate of WBF increase was similar for mating attempts that resulted in a copulation or rejection (Mann–Whitney U-test: *U*_(12, 21)_ = 109, *Z* = 0.63, *P* = 0.52) (Table 1, Fig. 3b). While females also increased their WBFs regardless of mating outcome, the rate of the frequency increase was significantly greater prior to a mating rejection compared to a copulation (Table 1, Fig. 3b) (Mann–Whitney U-test: *U*_(12, 21)_ = 24, *Z* = − 3.81, *P* < 0.01), owing to differences in the extent of the frequency increase (Mann–Whitney U-test: *U*_(12, 21)_ = 58, *Z* = − 2.54, *P* = 0.01) and the time interval of the increase (Mann–Whitney U-test: *U*_(12, 21)_ = 40.5, *Z* = 3.19, *P* < 0.01) (Table 1).

**Table 1:** Values of frequency interval (ΔF), time interval (Δt) and the rate of frequency increase (ΔF/Δt) during mating attempts

	Copulation (*n* = 12)	Mating rejection (*n* = 21)
ΔF (Hz)	♂: 91.2 ± 6.3	♂: 90.9 ± 4.9
	♀: 81.1 ± 10.3	♀: 111.6 ± 5.3
Δt (s)	♂: 0.325 ± 0.034	♂: 0.364 ± 0.025
	♀: 0.393 ± 0.048	♀: 0.220 ± 0.029
ΔF/Δt (Hz s^−1^)	♂: 314.68 ± 36.60	♂: 282.69 ± 27.35
	♀: 218.32 ± 29.59	♀: 655.52 ± 67.71

As we detected differences in the female rate of WBF increase between mate rejection or copulation subsequent to a mating attempt, we further analyzed this behavior. In females that rejected a male more than once (*n* = 18), we performed a paired comparison between two different mating attempts of the same female and found no differences in the rate of WBF increase (Wilcoxon matched pairs test: *Z* = 0.97, *P* = 0.32) (Additional file 1: Figure S2a). Thus, females consistently increased their WBFs during rejections. However, in females that rejected and then copulated with a male (*n* = 10), we performed a paired comparison between the copulation and a rejection of the same female and found significant differences in the rate of WBF increase between outcomes (Wilcoxon matched pairs test: *Z* = 2.84, *P* < 0.01) (Additional file 1: Figure S2b): the rate of increase was lower for mating attempts that resulted in copulation compared with a rejection. Thus, in our assays, the increasing rate of the female WBF predicted copulation or rejection. However, there are likely additional behavioral and physiological factors that influence mating.

### Male-male acoustic behaviors during free flight

We next analyzed acoustic and flying behaviors among groups of males. We introduced eight males into a soundproof chamber and made an audio record of their flight tones and a visual record of their flight characteristics. As only a portion of the introduced males flew at any given time, we have differing numbers of recordings of two, three and four males flying simultaneously. For our analysis, we selected 5 s intervals in which flying behavior was clear to analyze male flight trajectories using an ellipse [43], determining the distance of each individual in relation to the rest of the group and following the X and Y coordinates of each individual over this time frame (see “Methods”). To assess flight acoustics of male groups, we analyzed the average frequency difference of each group; the difference between the highest and lowest WBFs observed in the spectrogram represented the frequency bandwidth of the interaction among males during the 5 s segments analyzed.

**Fig. 4:**
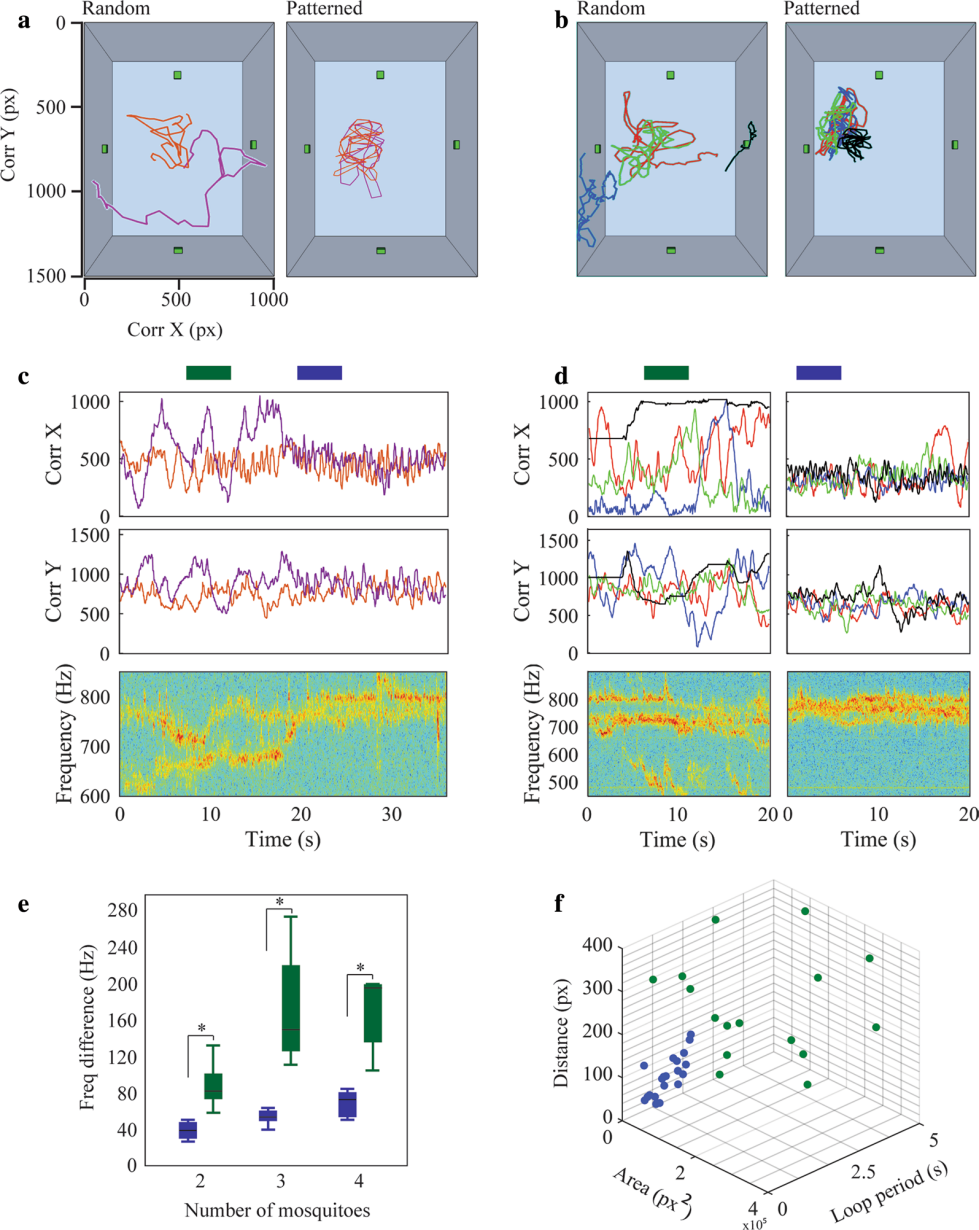
Acoustic and visual analysis of free-flying males. Two (**a**) and four (**b**) males in random flight (left panels) and patterned flight (right panels). Grey boxes represent the spatial distribution of the experimental arena. Green squares within the boxes indicate microphone location. Colored lines show the 2D trajectory for each male during 5 s recordings. The X (top) and Y (middle) coordinates of the flight trajectories and the high order spectrogram (bottom) of groups of two (**c**) and four (**d**) mosquitoes during random and patterned flight. Colored rectangles indicate the 5 s interval analyzed to associate flying pattern with acoustic behavior (green: random flight; blue: patterned flight). **e** Box and whiskers plots of the frequency difference of males flying randomly (green) and in patterns (blue). The box ranges from the first quartile to the third quartile of the distribution. A horizontal line across the box indicates the median. The whiskers extend from the quartiles to the extreme data points. Males flying randomly exhibit a higher frequency difference than males in patterned flight. **f** Differences in the characteristics of the flight trajectories of mosquitoes in random (green) or patterned flight (green)

Two distinct flying behaviors were apparent among groups of males flying simultaneously: random flight and a swarm-like, patterned flight (Fig. 4a, b; Additional file 3: Video S2), referred to hereafter as “patterned flight”. During random flight, male WBFs diverged or were distinct (Fig. 4c, d, bottom panels), but converged once patterned flight was initiated, reducing the frequency differences of the male flight tones (two males: Fig. 4c; four males: Fig. 4d; Additional file 3: Video S2). By examining flight behavior in 5 s intervals, we observed a significant change in the frequency difference between males in random flight and those in patterned flight. This change was consistent among groups of two (Mann–Whitney U-test: *U*_(8, 8)_ = 0, *Z* = − 3.36, *P* < 0.01), three (Mann–Whitney U-test: *U*_(5, 5)_ = 0, *Z* = − 2.61, *P* < 0.01) and four (Mann–Whitney U-test: *U*_(5, 5)_ = 0, *Z* = − 2.61, *P* < 0.01). The difference in WBF of groups in patterned flight was always smaller than groups in random flight (Fig. 4e). The mean frequency difference of groups of two, three and four males in patterned flight was 38.20 ± 3.33, 52.60 ± 4.01 and 68.21 ± 7.11 Hz, respectively. On the other hand, the mean frequency difference of groups of two, three and four males in random flight was 110.88 ± 19.91, 177.05 ± 31.16 and 167.65 ± 19.88 Hz, respectively.

During patterned flight, we observed that *An. albimanus* fly in an ellipsoid pattern around a central position, similar to what has been reported in *Cx. quinquefasciatus* [43]. Therefore, we quantified visual differences between random and patterned flight (where we observed acoustic interactions) by examining the trajectory of individual mosquitoes and analyzing the ellipsoid shape formed while flying [43]. We found significant differences in the characteristics of male trajectories between the two types of flight observed: the ellipse area representing random flight is significantly larger than the area representing patterned flight [random flight: (1.55 ± 0.29) × 10^5^ px^2^; patterned flight: (0.49 ± 0.06) × 10^5^ px^2^; Mann–Whitney U-test: *U*_(18, 18)_ = 58, *Z* = 3.24, *P* < 0.01]. We further measured the distance (in pixels) of each mosquito in relation to the rest of the group by calculating the average distance among the centroids of each ellipse in comparison with the others. We found that mosquitoes flying randomly are significantly less aggregated than males in patterned flight (random flight: 244.66 ± 21.03 px; patterned flight: 95.79 ± 11.15 px; Mann–Whitney U-test: *U*_(18, 18)_ = 69, *Z* = 2.89, *P* < 0.01). Finally, by separately analyzing the X and Y trajectories of males in flight, we observed characteristic loops (i.e. periodic movement) similar to those defined in previous studies [43]. Mosquitoes in patterned flight display loops with significantly shorter periods (random flight: 2.63 ± 0.39 s; patterned flight: 0.90 ± 0.08 s; Mann–Whitney U-test: *U*_(18, 18)_ = 33.5, *Z* = 4.03, *P* < 0.01) (Fig. 4f). Taken together, these data show that patterned flight is associated with distinctive acoustic behaviors.

## Discussion

Mosquito flight tones differ by species and are used to develop novel trapping tools [46] and recognition algorithms [47], highlighting the importance of flight tone characterization of disease vectors. While there is ample information about *Ae. aegypti*, *Cx. quinquefasciatus* and *An. gambiae* acoustic behavior prior to and during mating, there is no information about acoustic behavior of one the most important malarial vectors in the Americas, *An albimanus*. Furthermore, most acoustic characterization of mosquito vectors has been performed using immobilized specimens. Here, we characterized acoustic behavior of *An. albimanus.* We observed significant differences in the flight acoustics between free-flying and tethered *An. albimanus*, describe acoustic interactions between free-flying and tethered male–female couples, and identify a relationship between male-male acoustic interactions and the coordination of movement that occurs when males transition from random flight to patterned flight.

Tethered *An. albimanus* exhibit lower WBFs than other reported mosquito species (*Ae. aegypti* [4, 48], *An coluzzi* [40], *An. gambiae* [21, 22]). While WBFs vary among mosquito species [49], they can be affected by factors that include ambient temperature [4, 50], humidity and age [51]. We observed major acoustic differences between tethered and free-flying mosquitoes: (i) the mean WBF of free-flying *An. albimanus* is higher for both sexes; (ii) the mean WBF ratio between females and males (♀WBF/♂WBF) changes (tethered = 0.71, free flight = 0.61); and (iii) free-flying mosquitoes modulate their WBF to a greater extent than tethered individuals. Tethered insects have been used to study locomotion [52, 53], migration [52, 53] and wing movement [54]; these studies have demonstrated that tethering leads to unnatural flight behaviors and distorted wing strokes. Tethered individuals are restricted to a horizontal flight path, do not produce the equivalent lift required to achieve normal flight, and do not support their own body mass, factors that may give an inaccurate view of natural flight [55]. As flight tones are linked to motor function [56], our results show that restricting motion alters two flight tone characteristics: the WBF and the ability to modulate it. Although a decrease in the WBF linked to tethering has been reported for *Ae. aegypti* [4], *Cx. quinquefasciatus* [25], midges [44] and locusts [45], Villareal et al. [50] found no difference in WBF of tethered and free-flying *Ae. aegypti* females when assays were performed at the same temperature. Although our assays were performed at the same temperature, we found that tethering impacted *An. albimanus* WBFs.

Harmonic convergence, or the synchronization of frequencies between two specific harmonics during male–female interactions, has been described in *Ae. aegypti* [17, 18], *Cx. quinquefasciatus* [19] and *An. gambiae* [20, 21] using tethered individuals. This behavior appears to be related to the male auditory strategy used to track females [57] and, more recently, has been suggested to be a manifestation of male–female coordination prior to formation of the copula position [26]. We observed harmonic convergence in tethered *An. albimanus*. The time of convergence and the latency of the response were similar to *An. gambiae* [21] and the convergence events we detected have been observed in other species [18, 21, 37]. When examining male–female acoustic interactions during a mating attempt in free flight, we were unable to observe harmonic convergence using the criteria of Aldersley et al. [37]. Harmonic convergence presumably occurs rapidly during a mating attempt, resembling the early stages of *Ae. aegypti* precopulatory behavior (i.e. from the male approach to the end of RFM) [26]. It is possible that convergence similar to what we observed under tethered conditions occurs after male–female contact, as has been demonstrated in *Ae. aegypti* [26]. Thus, we might not have detected convergence in our assays as *An. albimanus* couples often did not exhibit long paired flight after making contact.

However, we observed a common precopulatory behavior in free flight: a rapid increase in male WBF immediately followed by a rapid increase in female WBF. A similar behavior is seen in *Cx. quinquefasciatus* [25], *An. coluzzii* and *An. gambiae* [40], where males increase their WBF in response to female flight tone. The rate of frequency increase in males was similar regardless of mating outcome, while a more rapid increase in female frequency resulted in mate rejection. It is possible that the frequency increase results from controlled flight to reach or escape a potential mate. Results from the *Culex* [25] and *Anopheles* [40] auditory system suggest that to locate females, males use the difference between their own frequency and that of the female [57] rather than the female WBF itself. The rapid change in female WBF during mate rejection immediately modifies the male–female WBF difference. Remaining within the optimal auditory sensitivity range may be a critical characteristic to reach and copulate with a female.

Male swarming behavior appears to be an obligatory feature of copulation for some *Anopheles* species [35, 58]. However, swarming behaviors have not been described for Latin American species. Furthermore, male-male interactions during swarming, and female mate selection within a swarm, are not well understood. Acoustic signaling might be an important factor during these interactions, although studies of male-male and male–female interactions in free flight are few. In our assays, *An. albimanus* males performed two identifiable types of flying behavior regardless of the number of males assessed: random and patterned flight. During random flight, males flew in large trajectories within the entire experimental arena. Once patterned flight was initiated, the large trajectories immediately gave way to a form of flight composed of small loops in a specific region of the arena. Interestingly, males in patterned flight acoustically interacted within a narrower band of frequency, linking flight pattern and acoustic behavior. This result is likely related to the clustering of male flight tones of closely located, tethered *Ae. aegypti* males [36]. During swarming, males must coordinate their flight patterns and recognize females that enter the swarm. It has been proposed that flight tones are used to coordinate movement during group flight [36], dividing into frequency clusters to reduce acoustic interference [59].

## Conclusions

Linking acoustic and flying behaviors under natural, unrestricted conditions will provide important information regarding behaviors during courtship and ultimately, female mate selection. Many of the male–female acoustic interactions we observed during mating attempts in free flight have been reported in other mosquito species, suggesting that such interactions are common during courtship in mosquito species and supporting the idea that acoustic interactions are crucial for successful copulation. This study also reveals a connection between male acoustics and flight characteristics, giving insight into the importance of male-male acoustic interaction during patterned flight and potentially giving clues about swarm formation and cohesion. Furthermore, *An. albimanus* males modulate their flight tones in the presence of other males. While this behavior likely entails additional sensory cues, targeting acoustic male interactions may disrupt essential reproductive behaviors of this, and potentially other non-swarming species. Control programmes that rely on the mass release of laboratory-reared mosquitoes [60–63] need to consider acoustic behavior, since the results presented here, as well as those of previous reports, demonstrate that different mosquito species display similar precopulatory acoustic behaviors, highlighting the importance of acoustics in mating success [27, 28]. Characterization of *An. albimanus* acoustic flight behaviors, an important Latin American malaria vector, is a step toward understanding male-male and male–female interactions prior to copulation and will aid in the improvement of vector surveillance and control programmes in areas affected by this species.

## Additional files

**Additional file 1: Figure S1.** The relationship between size and wingbeat frequency of **a** tethered and **b** free-flying mosquitoes recorded at 26 ± 2 °C. Linear regression between wing length and frequency of tethered males (blue, tethered: *R*^2^ < 0.01, Pearson’s *r* = 0.06, *P* = 0.58; free flight: *R*^2^ = 0.12, Pearson’s *r* = 0.35, *P* = 0.02) and females (red, tethered: *R*^2^ = 0.03, Pearson’s *r* = − 0.20, *P* = 0.05; free flight: *R*^2^ < 0.01, Pearson’s *r* = 0.03, *P* = 0.81). Although our rearing method did not intend to generate individuals of different sizes, the determination coefficient (*R*^2^) and Pearson’s correlation coefficient (*r*) shows that in males and females there is a weak or a non-significant relationship between size and wingbeat frequency. **Figure S2.** Paired comparison between **a** two rejections and **b** a rejection and a successful copulation of the same female. While there is no significant difference between rejections (Wilcoxon matched pairs test: *Z* = 0.97, *P* = 0.32), there is a significant decrease of the WBF rate of increase (Wilcoxon matched pairs test: *Z* = 2.84, *P* < 0.01) between rejections and successful copulations.

**Additional file 2: Video S1.** Video of male–female mating attempts: rejections and successful copulation. The left panel shows the experimental arena and the reconstruction of the flight trajectories of the male (in blue) and the female (in red). Right panels indicate the time variation of X (top) and Y (middle) coordinates of the flight trajectories and the spectrogram (bottom) of the fundamental frequency.

**Additional file 3: Video S2.** Comparison between random and patterned flight for groups of two and four mosquitoes. The left panel shows the experimental arena and the reconstruction of the flight trajectories of males. Right panels indicate the time variation of X (top) and Y (middle) coordinates of the flight trajectories and the spectrogram (bottom) of the fundamental frequency.

## Data Availability

The datasets generated during and/or analyzed during the present study are available from the corresponding authors upon reasonable request.

## Abbreviations

WBF: wing beat frequency
RFM: rapid frequency modulation
