# Supplementary figures and images for: Precopulatory acoustic interactions of the New World malaria vector Anopheles albimanus (Diptera: Culicidae)
Source: Parasit Vectors. 2019 Aug 1;12:386. doi: 10.1186/s13071-019-3648-8 (PMC6676525; doi:10.1186/s13071-019-3648-8)

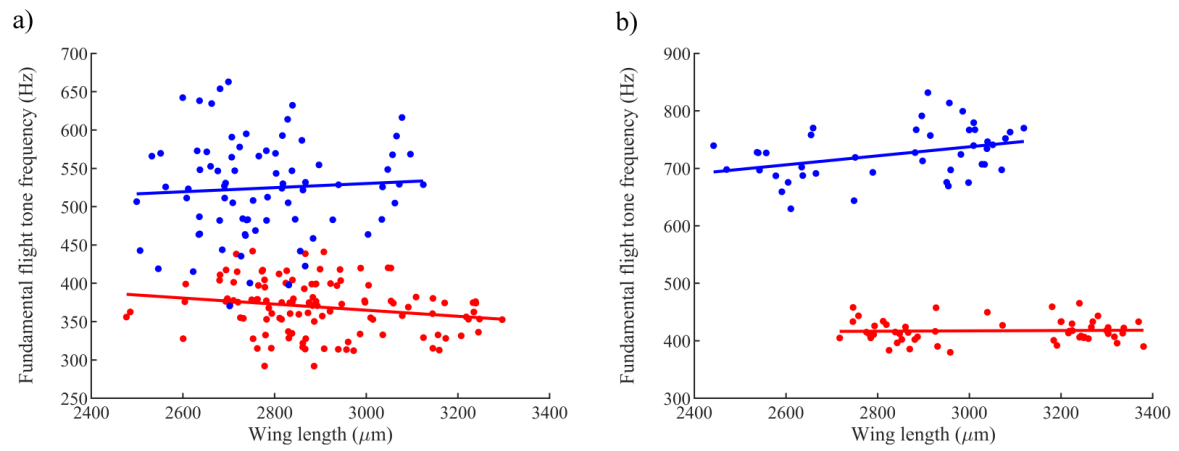

**Figure S1.**

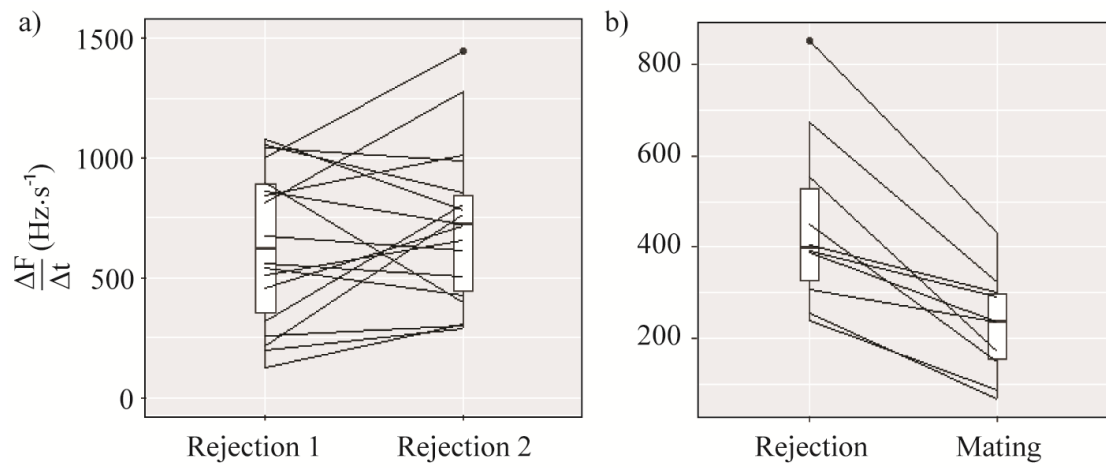

**Figure S2.**

Supplement: Supplementary file 1 — Additional file 1: Figure S1. The relationship between size and wingbeat frequency of a tethered and b free-flying mosquitoes recorded at 26 ± 2 °C. Linear regression between wing length and frequency of tethered males (blue, tethered: R2 < 0.01, Pearson’s r = 0.06, P = 0.58; free flight: R2 = 0.12, Pearson’s r = 0.35, P = 0.02) and females (red, tethered: R2 = 0.03, Pearson’s r = − 0.20, P = 0.05; free flight: R2 < 0.01, Pearson’s r = 0.03, P = 0.81). Although our rearing method did not intend to generate individuals of different sizes, the determination coefficient (R2) and Pearson’s correlation coefficient (r) shows that in males and females there is a weak or a non-significant relationship between size and wingbeat frequency. Figure S2. Paired comparison between a two rejections and b a rejection and a successful copulation of the same female. While there is no significant difference between rejections (Wilcoxon matched pairs test: Z = 0.97, P = 0.32), there is a significant decrease of the WBF rate of increase (Wilcoxon matched pairs test: Z = 2.84, P < 0.01) between rejections and successful copulations. [file 13071_2019_3648_MOESM1_ESM.pdf]
